# Multidrug-Resistant Bacterial Infections in Geriatric Hospitalized Patients before and after the COVID-19 Outbreak: Results from a Retrospective Observational Study in Two Geriatric Wards

**DOI:** 10.3390/antibiotics10010095

**Published:** 2021-01-19

**Authors:** Beatrice Gasperini, Antonio Cherubini, Moira Lucarelli, Emma Espinosa, Emilia Prospero

**Affiliations:** 1 Department of Biomedical Sciences and Public Health, Section of Hygiene, Preventive Medicine and Public Health, Università Politecnica delle Marche, 60126 Ancona, Italy; b.gasperini@univpm.it; 2 Geriatrics, Santa Croce Hospital, Azienda Ospedaliera Ospedali Riuniti Marche Nord, 61032 Fano, Italy; e.espinosa@univpm.it; 3 Geriatria, Accettazione Geriatrica e Centro di Ricerca Per L’invecchiamento, IRCCS INRCA, 60124 Ancona, Italy; a.cherubini@inrca.it (A.C.); m.lucarelli@inrca.it (M.L.)

**Keywords:** multidrug-resistant bacteria, COVID-19, *Staphylococcus*, older adults, hospitalization

## Abstract

The impact of the COVID-19 pandemic on multidrug-resistant (MDR) bacteria is unknown. The purpose of this study was to assess prevalence, etiology, and association with mortality of MDR bacteria in older adult patients before and after the first peak of the COVID-19 pandemic in Italy. An observational retrospective study was conducted in two geriatric wards of the Azienda Ospedaliera Ospedali Riuniti Marche Nord, Fano, and of the INRCA, IRCCS, Ancona, in the Marche Region, Italy, from December 2019 to February 2020 and from May to July 2020. A total of 73 patients (mean age 87.4 ± 5.9, 27.4% men) and 83 cultures (36 pre-COVID-19 and 47 post-COVID-19) were considered. Overall, 46 cultures (55.4%) reported MDR bacteria (50% in pre- and 59.6% in post-COVID-19 period, *p* = 0.384). MDR bacteria in bloodstream significantly increased in post-COVID-19 period (68.8% vs. 40.0% *p* = 0.038) and MDR bacteria in urine did not change (51.6 vs. 54.8%, *p* = 0.799). *Escherichia coli* was the main MDR bacterium in pre-COVID-19, *p* = 0.082 and post-COVID-19, *p* = 0.026. Among patients with MDR infection, in-hospital mortality was 37.5% and 68.8% in pre- and post-COVID-19, respectively (*p* = 0.104), and mortality at 30 days was higher in post-COVID-19 period (78.9% vs. 27.3%, *p* = 0.012). An increased number of MDR bacteria in bloodstream and mortality after MDR infection have been observed in the post-COVID-19 period.

## 1. Introduction

Currently, at least 700,000 people die each year due to MDR infections and this number could rise up to 10 million per year by 2050. A reduction in the effectiveness of antibiotics might increase exponentially the risk of medical and surgical procedures as well as immunosuppressive treatments, such as chemotherapy for cancer. Furthermore, the economic damage could be as catastrophic as the 2008–2009 global financial crisis [1]. The impact of COVID-19 pandemic on antimicrobial prescribing and multidrug-resistant (MDR) bacteria in the hospital setting is unknown [2].

Nonpharmacological behavioral changes that have been implemented during the COVID-19 pandemic to reduce the diffusion of SARS-CoV2 might also reduce the prevalence of MDR infections [3]. An improvement in hygiene practices in the hospital, use of personal protective equipment, antimicrobial soaps, and disinfectant cleaners have been adopted largely over the last few months, and these practices may reduce the spread of MDR. Besides, limitations in the number of people attending the hospitals and distance policies implemented for hospitalized patients can lead to future reduction of the circulation of bacteria.

Conversely, other aspects of the pandemic could determine a raise in the development of MDR. In particular, (i) cough and fever, which are the most prevalent symptoms of COVID-19, are independent factors associated with overuse of antibiotics in hospitals and communities [4]; (ii) the use of antimicrobials was largely prevalent in COVID-19 patients and more than 70% of them received antimicrobial treatment despite less than 10% had bacterial or fungal coinfections [5]; (iii) at the beginning of the pandemic, some broad-spectrum antimicrobial agents were suggested as treatments against COVID-19 [6,7] and were tested for a possible efficacy against SARS-CoV-2, i.e., teicoplanin, azithromycin, tetracycline [8]; (iv) excessive use of disinfectants and sanitizers could cause a rise in alcohol resistant bacteria [9].

Geriatric patients are more prone to developing infections. Ageing is associated with changes within the immune system and susceptibility to infection increases in association with comorbidities and medications. These patients are more susceptible to MDR infections due to physiological changes and comorbidity [10,11].

In the USA, the frequency of older people admitted to the hospital with resistant infections increased by 48.8% from 1997 to 2006 [12] and this trend has been confirmed for urinary tract infections in the following years. Patients with MDR infections have a higher likelihood to be discharged to other healthcare facilities, increased length of stay, hospital costs, and all-cause in-hospital mortality [13]. Therefore, older patients might be at higher risk of a possible increase in MDR infections secondary to the pandemic compared with younger patients.

In our study, we aimed to assess if the COVID-19 pandemic led to an increase of the infections sustained by MDR in hospitalized older patients.

The purposes of the present study were to assess the prevalence of MDR infections and the etiology of blood and urine infections in hospitalized older patients three months before and three months after the first peak of COVID-19 pandemic in Italy, and to evaluate their association with in-hospital mortality and mortality at 30 days.

## 2. Results

**Figure 1:**
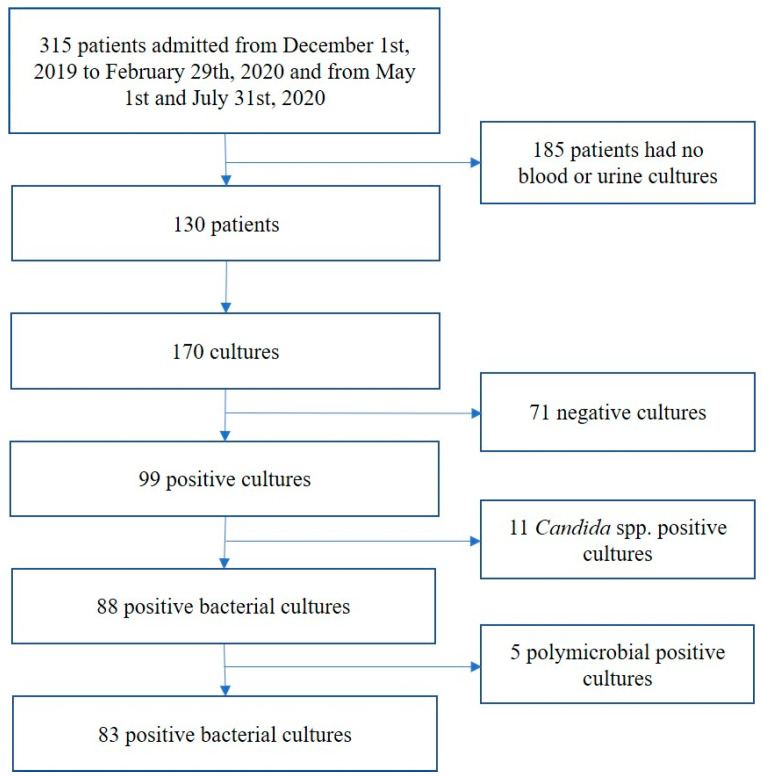
The flow chart of sample selection.

Data were extracted from the medical records of patients admitted during the period of the study. The flow chart of the patients’ selection is shown in Figure 1.

The total number of patients screened for inclusion was 315 and 130 of them were tested for a bloodstream or/and bacterial urinary tract infection. The final analysis included 73 subjects (33 pre-COVID-19 and 40 post-COVID-19).

A total of 170 cultures were taken (77 pre-COVID-19 and 93 post-COVID-19). There were 43 blood cultures (15 pre-COVID-19 and 28 post-COVID-19) and 127 urine cultures (62 pre-COVID-19 and 65 post-COVID-19). Overall, 71 cultures were negative (34 pre-COVID-19—10 blood and 24 urine; 37 post-COVID-19—12 blood and 25 urine). Among positive cultures, 11 were positive for *Candida* (four pre-COVID-19 and seven post-COVID-19) and five were polymicrobial (three pre and two post). Finally, 83 cultures were positive for bacterial infection (36 pre-COVID-19 and 47 post-COVID-19) and were analyzed.

### 2.1. Patients

Patients characteristics are shown in Table 1. Mean age was 87.4, patients admitted in the pre-COVID-19 period were slightly older, men were less than one-third, patients had severe disability (mean number of activities of daily living (ADL) preserved 1.2 + 1.6, 42.5% were bedridden), took 6.7 drugs per day, had a mean of 6.1 chronic conditions, the reason for admission was an infectious disease in 19 patients in pre-COVID-19 period (57.6%) and in 18 patients in post-COVID-19 period, and the mean length of stay did not differ in pre- and post-COVID-19 period.

### 2.2. Cultures

Among the 83 cultures positive for bacterial infections, 46 (55.4%) reported MDR infections (Table 2) and they represented 50% of the infections in pre-COVID-19 period (*n* = 18) and 59.6% in the post-COVID-19 period (*n* = 28) (*p* = 0.384).

Bloodstream infections were significantly increased in the post-COVID-19 period compared with pre-COVID-19 period (34.0% vs. 13.9%, *p* = 0.036). Conversely, the rate of urinary tract infections among the urine cultures was higher in pre-COVID-19 period (86.1% vs. 66.0%, *p* = 0.036). The MDR bloodstream infections significantly increased in the post-COVID-19 period (68.8% vs. 40.0%, *p* = 0.038), while MDR bacteria in urine did not change (51.6% vs. 54.8%, *p* = 0.799). Among MDR infections, in the post-COVID-19 period there was a decrease in the proportion of urine tract infections (60.7% vs. 88.9%, *p* = 0.038) and an increase in the proportion of bloodstream infections (11.1% vs. 39.3%, *p* = 0.038).

*Escherichia coli* (*E. coli*) infections were the most prevalent among total cultures (38.6%, *n* = 32), rising from 36.1% to 40.4% (*p* = 0.689) from the pre-COVID-19 to the post-COVID-19 period, respectively. Accordingly, the rate of *E. coli* MDR increased (69.2% vs. 78.9%, *p* = 0.105). The proportion of MDR bacteria was higher for *E. coli* than other bacteria in pre-COVID-19 period (*p* = 0.082) and in post-COVID-19 period (*p* = 0.026).

**Figure 2:**
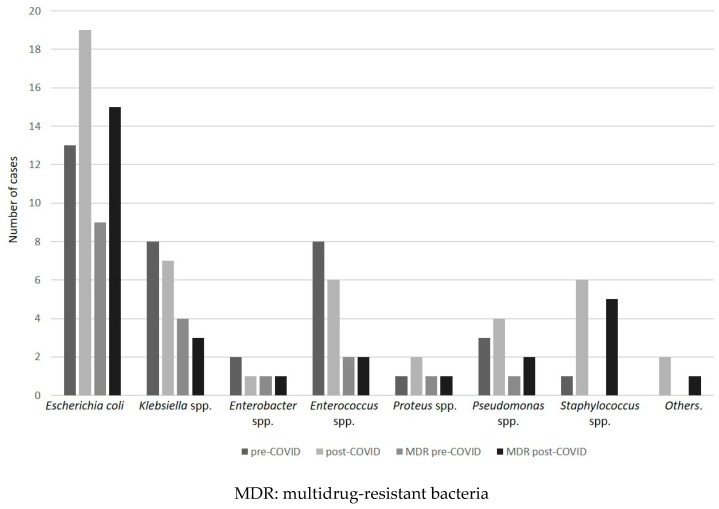
Number of total cases for each bacterium in accordance with the period (pre- and post-COVID-19) and number of MDR isolated bacteria in pre- and post-COVID-19 period.

The prevalence of *Staphylococcus* infections increased, raising from 1 in the pre-COVID-19 period to 6 in the post-COVID-19 (*p* = 0.132) with changing MDR rate, from 0% to 83.3% in pre- and post-COVID-19 period, respectively, (*p* = 0.058). No differences were found in the MDR infection prevalence for other bacteria (Table 2, Figure 2). Extended spectrum beta-lactamase (ESBL)-producing bacteria were the most prevalent MDR bacteria before and after the COVID-19 spread (41.7% and 38.3% in pre- and post-COVID-19 period, *p* = 0.705).

### 2.3. Mortality

Overall, 32.9% (*n* = 24) of the patients died during hospital stay. Mortality rate was 37.5% in patients with MDR infection in pre-COVID-19 period and 68.8% in post-COVID-19 period (*p* = 0.198).

After 30 days from discharge, of the 73 patients observed, 30 subjects died (41.1%) and 11 of them admitted during the pre-COVID-19 period and 19 in post-COVID-19 period (24.2% and 40.0%, respectively, *p* = 0.402) (Table 1). Considering only deaths in patients with MDR infections, three deaths were recorded in patients who had a MDR infection pre-COVID-19 and 15 in patients who had a MDR infection post-COVID-19 (27.3% vs. 79.0%, *p* = 0.012).

Differences emerged in mortality rate pre- and post-COVID-19 according to the MDR bacteria.

Mortality at 30 days was higher in subjects with MDR infection due to *Staphylococcus*: 60% of those with MDR *Staphylococcus* died compared to 26.8% of those with another MDR (*p* = 0.128). After 30 days from discharge, 100% of subjects with a previous infection due to MDR *Staphylococcus* died compared with 31.7% of subjects with MDR infection due to other bacteria (*p* = 0.003).

### 2.4. Antibiotics

During the hospitalization, 35 patients (48%) received at least two different antibiotics (in pre-COVID-19 period *n* = 14, 40.0% and in post-COVID-19 period *n* = 21, 60.0%, *p* = 0.391). Moreover, 5 out of 73 (6.9%) patients were treated with three antibiotics (100% in post-COVID-19 period), mainly with piperacillin/tazobactam (*n* = 18, 24.7%), ceftriaxone (*n* = 15, 20.5%), and meropenem (*n* = 12, 16.4%). Among patients who need a second antibiotic, levofloxacin (*n* = 9, 25.7%) and meropenem (*n* = 7, 20.0%) were mainly used. The mean duration of the antibiotic treatment was 5.7 ± 3.4 days (5.9 + 3.4 days in pre-COVID-19 period and 5.4 + 3.4 in post-COVID-19 period, *p* = 0.566) and 6.1 ± 3.1 days for the second antibiotic (6.2 + 2.6 in pre-COVID-19 period and 6.2 + 3.4 in post-COVID-19 period, *p* = 0.914).

Streptomycin and kanamycin had the highest rate of resistance (100%). Conversely, the rate of carbapenemase-producing bacteria was quite low. Finally, oxacillin demonstrated a resistance rate of 100% (Table 3). The resistance to imipenem, ampicillin, and sulbactam increased in post-COVID-19 period, although the difference was not statistically significant.

MDR infections were associated with the use of more than one antibiotic (48.6 vs. 66.7%) although this difference was not statistically significant (*p* = 0.104).

## 3. Discussion

Overall, 55.4% cultures reported MDR infections. The number of MDR bloodstream infections increased in the post-COVID-19 period and the number of urinary tract infections decreased. The proportion of MDR is higher for *E. coli* than for other bacteria in pre-COVID-19 and in post-COVID-19 period. Mortality at 30 days after MDR infection was higher in post-COVID-19 period and it was 100% in patients with a previous infection due to MDR *Staphylococcus.*

The overuse and inappropriate use of antibiotics are substantial contributors to MDR infections. COVID-19 might cause a rise in the inappropriate use of broad-spectrum antibiotics, which is one of the main causes for the development of MDR bacteria. The Center of Diseases Control (CDC) does not recommend the use of antibiotics during treatment of COVID-19 patients [14]. Despite this, it has been reported that up to 70% of COVID-19 patients received antibiotics, although only 10% had a bacterial superinfection [15]. Measures taken during the COVID-19 era could increase the long-term mortality due to the MDR bacteria and the impact of the pandemic on antimicrobial resistance should be elucidated [2,16].

During the influenza A virus pandemic, amoxicillin/clavulanate, macrolides, and broad-spectrum antibiotics such as levofloxacin and cephalosporins were largely used [17]. Studies during the H1N1 pandemic showed an increased number of infections due to *Streptococcus pneumoniae*, *Staphylococcus aureus*, *Streptococcus pyogenes*, and *Haemophilus influenzae.* The results of the present study are in accordance with these findings and an increased rate of *Staphylococcus* spp. infection was found in blood cultures in post-COVID-19 period.

Regarding urine infections, the data observed are consistent with previous studies that described the etiology of urinary MDR. Hospitalized older adults have a high rate of infections due to *Klebsiella* spp., *Enterococcus* spp., and *Pseudomonas* spp. [18]. A recent study by Folliero et al. analyzed the prevalence of urinary infections and the pattern of microorganisms involved and *Enterococcus faecalis* (12.9%) was the most isolated strain and among the Gram-negative bacteria, the *E. coli* (53.5%) [19]. The findings of this study demonstrated that *Enterococcus* spp. was a common bacterium detected in both pre- and post-COVID-19 periods and *E. coli* was the main pathogen in the post-COVID-19 period.

Folliero et al. described high resistance rates of trimethoprim-sulfamethoxazole (78.4%) and gentamicin (84.2%) [19]. In this study the resistance of streptomycin is 100% and 46.9% for trimethoprim-sulfamethoxazole, without any difference before and after the COVID-19 outbreak. The resistance of streptomycin is serious and worrying also in our study.

A report from the Italian Institute of Health of the national surveillance of bacteria producing carbapenemase highlight the widespread in Italy of carbapenemase-producing *Enterobacteriaceae*, especially in hospitalized patients. In 2019 the incidence of reported cases increased compared to the previous three-year period. Central Italy, including the Marche region, is the area with the highest incidence of reported cases and together with southern Italy showed an increased incidence rate compared to 2018. The most common pathogen was *Klebsiella pneumoniae* kpc (carbapenemase-producing *Klebsiella pneumoniae*). In our study the rate of carbapenem resistance involved 19.5% MDR cultures. The most worrying finding in our study was the rate of extended spectrum beta-lactamase, which is responsible for a half of MDR resistant bacteria [20].

Previous Italian studies demonstrated that *E. coli* was the most common microorganism isolated in urinary culture of older adults hospitalized [21]. Our data are consistent with these findings. Vincitorio et al. described a rate of 27% of isolated *Escherichia* spp. [22]. We found a rate of 36.1% among the pre-COVID-19 period that increased up to 40.4% in the post-COVID-19 period. Nonetheless, in our study the proportion of MDR was higher for *E. coli* than for other bacteria in pre-COVID-19 (*p* = 0.082) and in post-COVID-19 periods (*p* = 0.026). The infections due to the MDR bacteria were associated with an increased mortality rate within 30 days after discharge, in accordance with previous findings [18,23].

Intensive care unit studies in Italy demonstrated an increase in the rate of infections by *Klebsiella* carbapenemase-resistant, *E. coli*, and *Enterococcus* spp., but these studies considered both COVID-19 and no COVID-19 patients [24,25]. Conversely, in a Spanish study carried out in a tertiary hospital for 20 weeks before and during the COVID-19 spread, the mean incidence density of blood infections caused by MDR organisms remained stable, with a value of 0.36 ± 0.42 cases per 1000 occupied bed days in the COVID-19 period and 0.33 ± 0.28 bloodstream infections per 1000 occupied bed days in the period before the national lockdown [26]. Our findings are different due to the increased rate of bloodstream infection positive for *Staphylococcus* MDR. Probably, there are differences related to the setting of care: patients are more susceptible to multidrug-resistant microorganisms in intensive care units than in medical wards [1].

Finally, a decrease of the urinary tract infections has been observed. Although data are not available for the present sample, it is well-known that during hospitalization it may be necessary to place a urinary catheter. It is possible that an increased use of personal protective equipment i.e., gloves, mask, and gowns, and a reinforcement in the disinfection procedures may have had an impact on improving the insertion and maintenance procedure of the urinary catheter.

The main limitation of the present study is the small number of patients involved. However, we analyzed a subset of hospitalized patients at high risk of adverse outcomes for infections and a comparison was done to compare data before and after the first COVID-19 peak in one of the most involved Italian regions. A second limitation is the number of cultural examinations. Not all patients with infection underwent cultural examinations and empirical therapy is sometimes administered. In addition, in some cases a culture could result negative although the patient had an infection (i.e., a culture of urine in patients who were already treated with an antibiotic could result negative although there is an infection). Despite these limitations, studies are needed to investigate the spread of MDR infections in post-COVID-19 period compared with the pre-COVID-19 period and our study is aimed to address this topic in a population of older patients at very high risk of complications due to the MDR infections.

## 4. Materials and Methods

An observational retrospective study was conducted among a convenience sample of patients admitted to the Geriatric Unit of the Azienda Ospedaliera Ospedali Riuniti Marche Nord, Fano (22 beds), and the Geriatric Unit of the INRCA, IRCCS, Ancona (24 beds), from 1 December 2019 to 29 February 2020 and from 1 May and 31 July 2020, located in the Marche Region, Italy.

In the Marche Region, the first case of COVID-19 was diagnosed on 26 February 2020. From this date, a rapid increase in the number of COVID-19 patients forced the assignment of a growing number of beds and staff members to treat these patients. Consequently, some internal medicine and geriatric wards were converted into COVID-19 Units. The highest diffusion of the virus was recorded in the North of the Marche Region (Pesaro and Ancona provinces) and the peak of the first wave of the infection was reached in the middle of March. At the beginning of May, it was possible to gradually reopen geriatric wards to treat non-COVID-19 patients.

Between 1 March 2020 and 30 April, the wards selected for this study were closed and the staff members were assigned to COVID-19 departments. For the purposes of our study we analyzed data from the three months before and after the closure due to the pandemic. During the period of the study, patients were admitted into the two wards for other clinical reasons than COVID-19. The COVID-19 infection was excluded with a nasopharyngeal swab and a chest X-ray, as well as clinical examination, before the admission to the ward.

### 4.1. Inclusion and Exclusion Criteria

Patients with at least one culture (blood or urine) collected during the hospitalization (from the admission in the Emergency Department until discharge) were screened. Only patients with a positive culture for bacteria were included in the study. Exclusion criteria were as follows: patients without any culture during hospital stay; patients whose urinary or blood cultures were negative (number of colony forming units per mL (CFU/mL) less than 100); patients with *Candida* spp. infections; patients with samples considered to be contaminated (if the growth of two or more bacterial species was observed).

### 4.2. MDR

Antimicrobial resistance was defined according to breaking points recommended by the European Committee on Antimicrobial Susceptibility Testing (EUCAST) [27]. The antibiotics examined in this study were ampicillin, ampicillin/sulbactam, amoxicillin/clavulanic acid, amikacin, cefotaxime, cefuroxime, fosfomycin, gentamicin, ciprofloxacin, imipenem, levofloxacin, trimethoprim/sulfamethoxazole, tobramycin, piperacillin/tazobactam, erythromycin, fusidic acid, cefoxitin, linezolid, teicoplanin, vancomycin, clindamycin, oxacillin, and daptomycin. Methicillin-resistance, extended-spectrum betalactamase (ESBL) and carbapenemase production were recorded. MDR was defined as nonsusceptibility to at least one agent in three or more antimicrobial categories [28].

### 4.3. Patient Variables

For each patient the following variables were recorded: age, gender, number of chronic diseases, drugs taken every day, functional status (assessed using Activities of Daily Living and Instrumental Activities of Daily Living scales), mobility, antibiotics allergies, and antibiotic treatment during hospitalization (type, dose, and duration). Administrative data were also recorded, such as date of hospital admission, date of discharge, length of stay, and type of discharge (home, facility outside the hospital, death). A follow-up at 30 days was performed, obtaining data from medical records or electronic database (i.e., laboratory database). Patients whose follow-up data were not available were not considered as “unknown” in the analysis.

### 4.4. Ethical Approval

The Ethics Committee of Marche Region does not require formal approval for observational studies that do not involve the use of drugs. The work was carried out in accordance with The Code of Ethics of the World Medical Association for experiments involving humans (Declaration of Helsinki) and research on health databases (Declaration of Taipei). Patients and caregivers gave their written consent to use their personal data at the admission into the Hospital. Anonymity of patients was guaranteed during the whole process of data analysis and results reporting.

### 4.5. Statistical Analysis

A descriptive analysis of the patient’s characteristics was performed. Categorical variables were presented as number and percentage. Continuous variables were presented as mean and standard deviation or median and range, as appropriate. The type and the resistance of isolated bacteria were described, as well as the rate of the resistance for any antibiotics. A description of the antibiotic used in our sample was proposed, including the type of antibiotic chosen and the duration of therapy, in days. Comparisons were made using chi-square test for categorical variables or independent samples *t*-test for continuous data. The significance was posed at <0.05. Data were analyzed using IBM SPSS software (version 25.0; IBM SPSS Inc., New York, NY, USA).

## 5. Conclusions

In conclusion, we identified an increased number of bloodstream infections and an increased mortality after MDR infection in post-COVID-19 period. *E. coli* has the higher rate of MDR, especially in the post-COVID-19 period. Bloodstream infections due to *Staphylococcus* in the post-COVID-19 period were associated with a higher mortality rate at 30 days after discharge. Future studies could clarify the real rate of bacterial coinfection in COVID-19 patients and might establish the benefit derived from appropriate antimicrobial therapy in those patients.

## Figures and Tables

**Table 1:** Patients characteristics according to the period of hospital admission (pre-COVID-19, from 1 December 2019 to 29 February 2020 and post-COVID-19, from 1 May and 31 July 2020).

	Total Sample N = 73	Pre-COVID-19 N = 33	Post-COVID-19 N = 40	*p*
**Age (mean ± SD)**	87.4 ± 5.9	88.8 ± 5.9	86.1 ± 5.7	0.054
**Male (N, %)**	20 (27.4)	8 (24.2)	12 (30.0)	0.532
**Functional status (mean ± SD)**				
ADL preserved	1.2 ± 1.6	1.3 ± 1.5	1.1 ± 1.7	0.461
IADL preserved	0.4 ± 1.2	0.5 ± 1.3	0.3 ± 1.1	0.662
**Mobility (N, %)**				0.934
Bedridden	31 (42.5)	14 (42.4)	17 (42.5)	
Bed-chair	19 (26.0)	8 (24.2)	11 (27.5)	
Walking aid	20 (27.4)	10 (30.3)	10 (25.0)	
Autonomous	3 (4.1)	1 (3.0)	2 (5.0)	
**Drugs taken daily (mean ± SD)**	6.7 ± 2.8	6.3 ± 3.3	7.2 ± 2.3	0.220
**Chronic diseases (mean ± SD)**	6.1 ± 2.0	6.1 ± 2.2	6.1 ± 1.9	0.989
**Reason for admission**				0.188
Infectious disease (N, %)	37 (50.7)	19 (57.6)	18 (45.0)	
Other reasons (N, %)	36 (49.3)	14 (42.4)	22 (55.0)	
**Length of stay, days (mean ± SD)**	12.3 ± 7.0	11.4 ± 5.8	13.0 ± 7.9	0.318
**In-hospital mortality (N, %)**	24 (32.9)	8 (24.2)	16 (40.0)	0.154
**among MDR infections**	14 (58.3)	3 (37.5)	11 (68.8)	0.198
**30-days mortality (N, %)**	30 (41.1)	11 (33.3)	19 (47.5)	0.402
**among MDR infections**	18 (60.0)	3(27.3)	15 (78.9)	0.012

SD: standard deviation; ADL: activities of daily living. IADL: instrumental activities of daily living. MDR: multidrug-resistant.

**Table 2:** Characteristics of cultures positive for bacterial infection taken in pre-COVID-19 and post-COVID-19 period.

	Total Positive Cultures N = 83	Pre-COVID-19 N = 36	Post-COVID-19 N = 47	*p*
**Type of culture** **(N, %)**				
Urinoculture	62 (74.7)	31 (86.1)	31 (66.0)	0.036
Urinoculture with MDR	33 (53.2)	16 (51.6)	17 (54.8)	0.799
Bloodstream culture	21 (25.3)	5 (13.9)	16 (34.0)	0.036
Bloodstream with MDR	13 (61.9)	2 (40.0)	11 (68.8)	0.038
**MDR infections** **(N, %)**	46 (55.4)	18 (50.0)	28 (59.6)	0.384
**MDR bacteria** **(N, %)**				0.956
*E. coli*	32 (38.6)	13 (36.1)	19 (40.4)	0.689
MDR	24 (75)	9 (69.2)	15 (78.9)	0.105
*Klebsiella* spp.	15 (18.1)	8 (22.2)	7 (14.9)	0.390
MDR	7 (46.6)	4 (50.0)	3 (42.8)	0.289
*Enterococcus* spp.	14 (16.9)	8 (22.2)	6 (12.8)	0.254
MDR	4 (28.6)	2 (25)	2 (33.3)	0.401
*Proteus* spp.	3 (3.6)	1 (2.8)	2 (4.2)	0.721
MDR	2 (66.7)	1 (100)	1 (50)	0.747
*Pseudomonas* spp.	7 (8.4)	3 (8.3)	4 (8.5)	0.977
MDR	3 (42.8)	1 (33.3)	2 (50)	0.831
Others	2 (2.4)	0 (0)	2 (4.2)	0.210
MDR	0 (0)	0 (0)	0 (0)	-
*Enterobacter* spp.	3 (3.6)	2 (5.6)	1 (2.1)	0.407
MDR	1 (33.3)	1 (50)	0 (0)	0.207
*Staphylococcus* spp.	7 (8.4)	1 (2.8)	6 (12.8)	0.132
MDR	5 (71.4)	0 (0)	5 (83.3)	0.058
**MRSA**	1 (1.2)	0 (0)	1 (2.1)	0.482
**ESBL**	33 (39.8)	15 (41.7)	18 (38.3)	0.705
**CPB**	9 (10.8)	3 (8.3)	6 (12.8)	0.725

MDR: multidrug-resistant bacteria. MRSA: Methicillin-resistant *Staphylococcus aureus*. ESBL: extended spectrum beta-lactamase producing bacteria. CPB: carbapenemase-producing bacteria.

**Table 3:** Number and percentage of resistance reported for each antibiotic. The percentage is referred to the total number of antibiograms including the antibiotic.

	Total Positive Cultures N = 83	Pre-COVID-19 N = 36	Post-COVID-19 N = 47	*p*
Amikacin	6 (10.0)	3 (10.7)	3 (9.4)	0.539
Amoxicillin/clavulanic	23 (46.9)	11 (44.0)	12 (50.0)	0.674
Ampicillin	3 (27.3)	1 (16.7)	2 (40.0)	0.387
Ampicillin/sulbactam	3 (27.3)	1 (16.7)	2 (40.0)	0.387
Cefepime	16 (51.7)	5 (71.4)	11 (45.8)	0.259
Cefotaxime	36 (63.2)	17 (68)	19 (59.4)	0.503
Ceftazidime	32 (52.5)	15 (53.6)	17 (51.6)	0.384
Ciprofloxacin	45 (64.3)	24 (75)	21 (55.3)	0.860
Clindamycin	5 (71.4)	1 (100)	4 (66.7)	0.495
Erythromycin	4 (57.1)	1 (100)	3 (50.0)	0.999
Ertapenem	7 (12.3)	3 (12)	4 (12.5)	0.954
Fosfomycin	12 (22.2)	5 (20.8)	7 (23.3)	0.826
Fusidic acid	2 (28.6)	0 (0)	2 (33.3)	0.999
Gentamicin	28 (35.9)	12 (34.3)	16 (37.2)	0.789
Imipenem	3 (27.3)	1 (16.7)	2 (40.0)	0.387
Kanamycin	13 (100)	6 (100)	7 (100)	-
Levofloxacin	12 (80)	4 (80)	8 (80)	0.999
Linezolid	1 (5.3)	0 (0)	1 (8.3)	0.999
Meropenem	5 (8)	2 (7.4)	3 (8.5)	0.179
Nitrofurantoin	1 (4.0)	1 (8.3)	0 (0)	0.288
Oxacillin	7 (100)	1 (100)	6 (100)	-
Piperacillin/tazobactam	16 (25.4)	9 (32.1)	7 (20.0)	0.271
Rifampicin	2 (28.6)	0 (0)	2 (33.3)	0.999
Streptomycin	9 (100)	4 (100)	5 (100)	-
Teicoplanin	3 (16.7)	1 (14.3)	2 (18.2)	0.999
Tetracycline	4 (57.1)	1 (100)	3 (50.0)	0.233
Tmt/sulfamethoxazole	30 (46.9)	15 (57.7)	15 (39.5)	0.118
Tigecycline	2 (3.9)	1 (5.3)	1 (3.1)	0.704
Vancomycin	2 (12.5)	1 (20)	1 (9.1)	0.999

## Data Availability

Restrictions apply to the availability of these data. Data are available from the authors with the permission of Azienda Ospedaliera Ospedali Riuniti Marche Nord and INRCA-IRCCS.
